# Light‐Activated Electron Transfer and Catalytic Mechanism of Carnitine Oxidation by Rieske‐Type Oxygenase from Human Microbiota

**DOI:** 10.1002/anie.202012381

**Published:** 2020-12-28

**Authors:** Muralidharan Shanmugam, Mussa Quareshy, Alexander D. Cameron, Timothy D. H. Bugg, Yin Chen

**Affiliations:** ^1^ Manchester Institute of Biotechnology (MIB) & Photon Science Institute (PSI) University of Manchester 131 Princess Street Manchester M1 7DN UK; ^2^ School of Life Sciences University of Warwick Gibbet Hill Road Coventry CV4 7AL UK; ^3^ Department of Chemistry University of Warwick Gibbet Hill Road Coventry CV4 7AL UK

**Keywords:** annealing, electron paramagnetic resonance, iron-sulfur proteins, metalloenzymes, redox enzyme

## Abstract

Oxidation of quaternary ammonium substrate, carnitine by non‐heme iron containing Acinetobacter baumannii (Ab) oxygenase CntA/reductase CntB is implicated in the onset of human cardiovascular disease. Herein, we develop a blue‐light (365 nm) activation of NADH coupled to electron paramagnetic resonance (EPR) measurements to study electron transfer from the excited state of NADH to the oxidized, Rieske‐type, [2Fe‐2S]^2+^ cluster in the AbCntA oxygenase domain with and without the substrate, carnitine. Further electron transfer from one‐electron reduced, Rieske‐type [2Fe‐2S]^1+^ center in AbCntA‐WT to the mono‐nuclear, non‐heme iron center through the bridging glutamate E205 and subsequent catalysis occurs only in the presence of carnitine. The electron transfer process in the AbCntA‐E205A mutant is severely affected, which likely accounts for the significant loss of catalytic activity in the AbCntA‐E205A mutant. The NADH photo‐activation coupled with EPR is broadly applicable to trap reactive intermediates at low temperature and creates a new method to characterize elusive intermediates in multiple redox‐centre containing proteins.

## Graphical abstract

Using photoactivated NADH and EPR spectroscopy, a method to monitor the changes in redox state(s) of the metal centers in the multi‐component, non‐heme enzymes was developed. Combined with annealing, the detailed mechanism of the catalytic conversion of carnitine into TMA and MSA by *Ab*CntA/*Ab*CntB in the human microbiota are investigated.

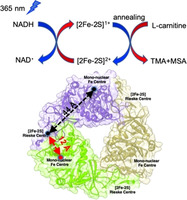

Rieske‐type oxygenases catalyze a wide range of reactions in nature, including oxidation of quaternary amines to trimethylamine (TMA).[^1^, ^2^, ^3^] The formation of TMA and its subsequent oxidation by hepatic flavin‐containing oxygenases to trimethylamine‐oxide (TMAO) by human gut microbiota promotes cardiovascular diseases.[^4^, ^5^, ^6^] Several quaternary ammonium oxygenases have been identified in the last decade, and studies of their active‐sites have been reported.[^7^, ^8^, ^9^] These enzymes comprise a reductase protein containing ferredoxin domain or Rieske [2Fe‐2S]^2+^ cluster, and an oxygenase protein containing a non‐heme, mononuclear iron center (monoFe).[^8^, ^10^] The carnitine oxygenase (CntA) and reductase (CntB) from *Acinetobacter baumannii* convert carnitine into TMA and malic‐semi‐aldehyde (MSA).^[9]^


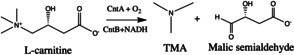


The NADH reductase domain (*Ab*CntB) contains a flavin mononucleotide (FMN) binding site and a plant‐type ferredoxin, [2Fe‐2S]^2+^ cluster.[^1^, ^2^, ^9^, ^11^, ^12^, ^13^] The oxygenase domain (*Ab*CntA‐WT) is an α_3_ homo‐trimer and contains the monoFe site and a Rieske‐type, [2Fe‐2S]^2+^ cluster in each α‐subunit with a head‐to‐tail interaction between each monomer.^[11]^ The recent crystal structure of *Ab*CntA with (PDB code: 6Y8J)^[9B]^ and without carnitine (PDB code: 6Y9D)^[9B]^ shows that these two redox centers are separated by 44 Å within a α‐subunit, so it is proposed that the electron transfer occurs across the neighboring subunits separated by ≈12 Å (Figure 1), via an electron transfer pathway containing bridging glutamate E205.^[9B]^ The mutation of E205 (E205A, E205Q and E205D) causes loss of catalytic activity.^[9]^ Previous bio‐physical studies on the Rieske‐type oxygenases demonstrated that reductase domain mediates electron‐transfer between the reductase and oxygenase domains, and the subsequent catalysis takes place at the monoFe.[^11^, ^12^, ^13^] The conversion of carnitine into TMA and MSA involves two single‐electron transfer processes from *Ab*CntB to *Ab*CntA.

**Figure 1:**
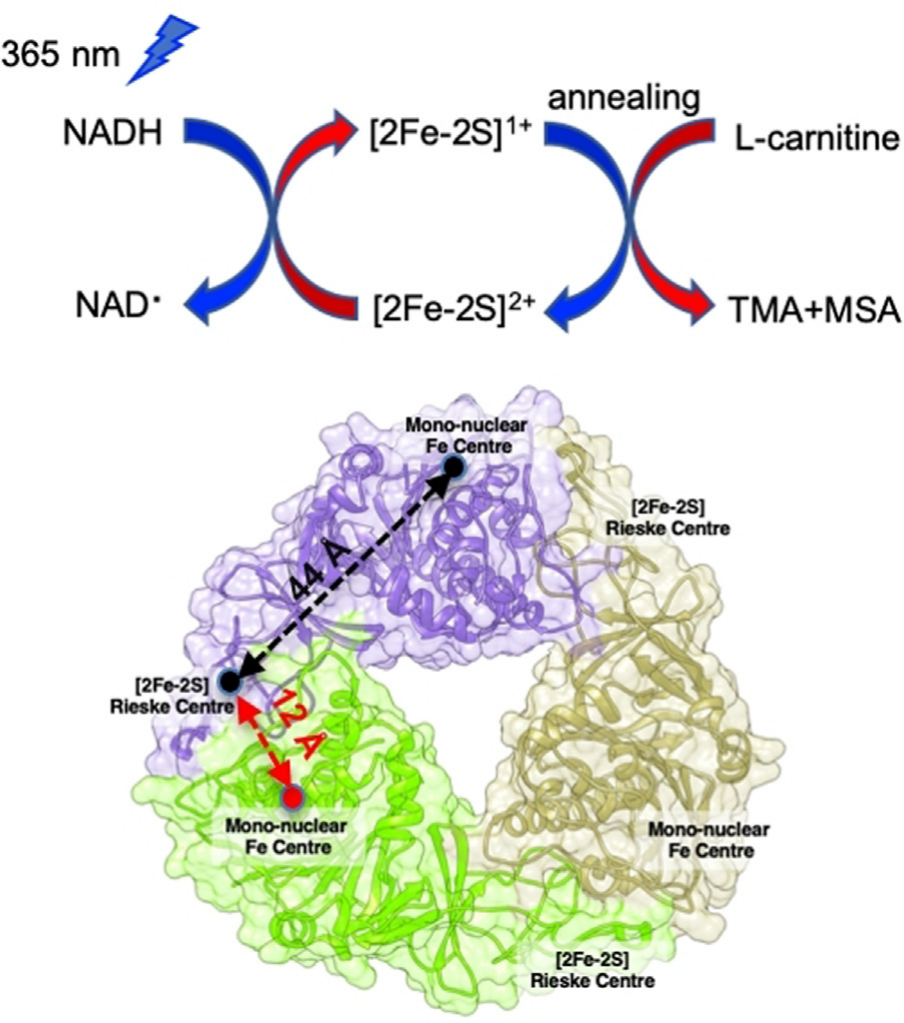
Schematic of NADH photoactivation coupled with EPR used to monitor the electron transfer from NADH to the Rieske‐type, [2Fe‐2S]^2+^ in *Ab*CntA variants (e.g. *Ab*CntA; PDB ID: 6Y8J)^[9b]^ and subsequent, carnitine catalysis takes place at the monoFe (where EPR‐silent, ferrous center is converted into high‐spin, ferric species; *S*=5/2) when the photo‐reduced, *Ab*CntA‐WT sample was annealed at higher temperatures (more details; see Figure 4).

Owing to the involvement of multi‐component redox centers, spectroscopic characterization of active intermediates involved in the carnitine oxidation is challenging. To reduce the number of redox‐centers, we developed a method to use photoactivated NADH to initiate the carnitine catalytic cycle by injecting a single electron, in combination with EPR spectroscopy, which is applicable to other complex metalloenzymes.

The photo‐excitation of NADH at 355 nm leads to the formation of solvated electrons, as well as radical species, which are effective in reducing the primary redox center in the fluid/frozen solution of protein (e.g. RT, 240 K or 77 K).[^14^, ^15^] It is well established in laser flash‐photolysis to probe the electron transfer kinetics in metalloenzymes by following changes in the absorption spectrum.[^16^, ^17^] This methodology has never been coupled with EPR to monitor the electron transfer process and subsequent redox changes occur in metalloenzymes. This method is similar to cryolitic reduction (^60^Co source of γ‐irradiation) experiments, where the electron injection converts the EPR‐silent metalloproteins into EPR‐active or *vice versa*.[^18^, ^19^] However, the number of EPR active species produced after the photoactivation of NADH is far less compared to the cryolitic reduction method.^[14]^ The advantage of coupling NADH photoactivation with EPR is that both redox processes, (i) EPR‐active to EPR‐silent and (ii) EPR‐silent to EPR‐active could be probed even for multi‐component redox centers. In contrast laser flash photolysis predominantly follows only a single redox‐center absorption features.[^16^, ^17^] By annealing the frozen protein solution at higher temperatures, the transient/elusive intermediates are trapped and spectroscopically characterized by EPR if the intermediates are EPR active.^[19]^ The applications of cryolytic reduction and subsequent annealing have been successfully demonstrated for a number of heme enzymes.[^18^, ^19^, ^20^, ^21^]

In this study, as a proof‐of‐principle, NADH‐photoactivation coupled with EPR is used for a diamagnetic, *Ab*CntA‐WT oxygenase enzyme to monitor the electron transfer process to the Rieske, [2Fe‐2S]^2+^ center and subsequent carnitine oxidation at monoFe. Similar studies were also performed on the *Ab*CntA‐E205A mutant. Here, we used a blue‐light (365 nm), NADH photoactivation at 240 K to initiate the electron transfer. Using annealing and EPR spectroscopy, the initial reduction of the Rieske center and subsequent catalysis at monoFe are monitored. Initially, we performed studies on ‘substrate‐free and substrate bound“ *Ab*CntA WT oxygenase enzyme in the presence of NADH, for which solution properties have been reported.^[9]^

The *Ab*CntA‐WT remains in its ferrous state when exposed to the air. The EPR spectra of *Ab*CntA‐WT in the presence and absence of NADH show residual EPR signals (Figure 2; black and red traces; top and bottom panels) from the one‐electron reduced Rieske center. This is consistent with predominant (>95 %) oxidized state for the Rieske center and the monoFe as a ferrous oxidation state (Figure S7; wide‐swept, cw‐EPR, top panel), as reported for other Rieske‐type oxygenases.[^12^, ^13^] This is in contrast to a recent report, where a high‐spin, *S*=5/2, ferric oxidation state is observed for *Ab*CntA.^[36]^ It is unclear whether this is due to the autooxidation of the non‐heme iron center. The observation of identical EPR spectra before and after the addition of NADH to *Ab*CntA‐WT implies that NADH has no effect on the electronic structure of the redox centers prior to photoactivation (Figure 2; black and red traces; top and bottom panel). This implies that electron transfer from NADH to the Rieske center is normally mediated through the reductase domain of *Ab*CntB.^[9]^ However, photoactivation of NADH at 240 K for 10 minutes led to the formation of one‐electron reduced Rieske center in *Ab*CntA‐WT (Figure 2; blue traces; top and bottom panels). Similar experiments in the presence of carnitine (*Ab*CntA‐WT+carnitine+NADH) also led to one‐electron reduced Rieske center but with slightly altered spin‐Hamiltonian parameters, specifically along g_3_. The intensities of the EPR signals continuously grow when the samples are photoactivated for longer times at 240 K (Figure S1; top). It is observed that the intensities of the EPR signals reached near saturation after 30 min of photoactivation (Figure S1; top). The photoactivation of *Ab*CntA‐WT+carnitine in the absence of NADH for up to 120 min shows no formation of one‐electron reduced Rieske center, demonstrating that photoexcited NADH acts as an one‐electron donor to reduce the Rieske center (Figures 1; middle panel, blue trace and S1; bottom panel). This is consistent with previous reports.[^16^, ^17^] The comparisons of EPR spectra of *Ab*CntA‐WT reduced by excess dithionite and by photoactivated NADH shows more reduction of the Rieske center by NADH‐photoactivation (Figures S9–S12). The g_3_ component of the observed EPR signals has been shifted towards low‐field in the presence of carnitine (Figure 1; double header, dotted magenta arrow). The spectrum shows significant broadening along the g_1_ region, including a shift towards upfield in the g_2_ component of the rhombic, g‐ tensor (Figure S2). All these changes and line broadening indicate overlapping of two different EPR‐active species; a one electron reduced Rieske center and a plausibly new EPR‐active species, *S*=1/2 at the monoFe. It is also possible that the electronic structure of the reduced Rieske center is altered upon the substrate binding, which accounts for the second EPR species overlapped in this region. The EPR spectra of *Ab*CntA‐WT in the presence and absence of carnitine were successfully simulated (Figures S3 and S4) using the spin‐Hamiltonian parameters provided in Table S1]. The observed g‐tensor, [2.016 1.906 1.793] for species 1 is significantly different to that reported in the literature for the ferric(hydro)peroxo/Fe^V^‐oxo species and is likely due to the oxygen/nitrogen rich ligand field at the monoFe.[^28^, ^29^]

**Figure 2:**
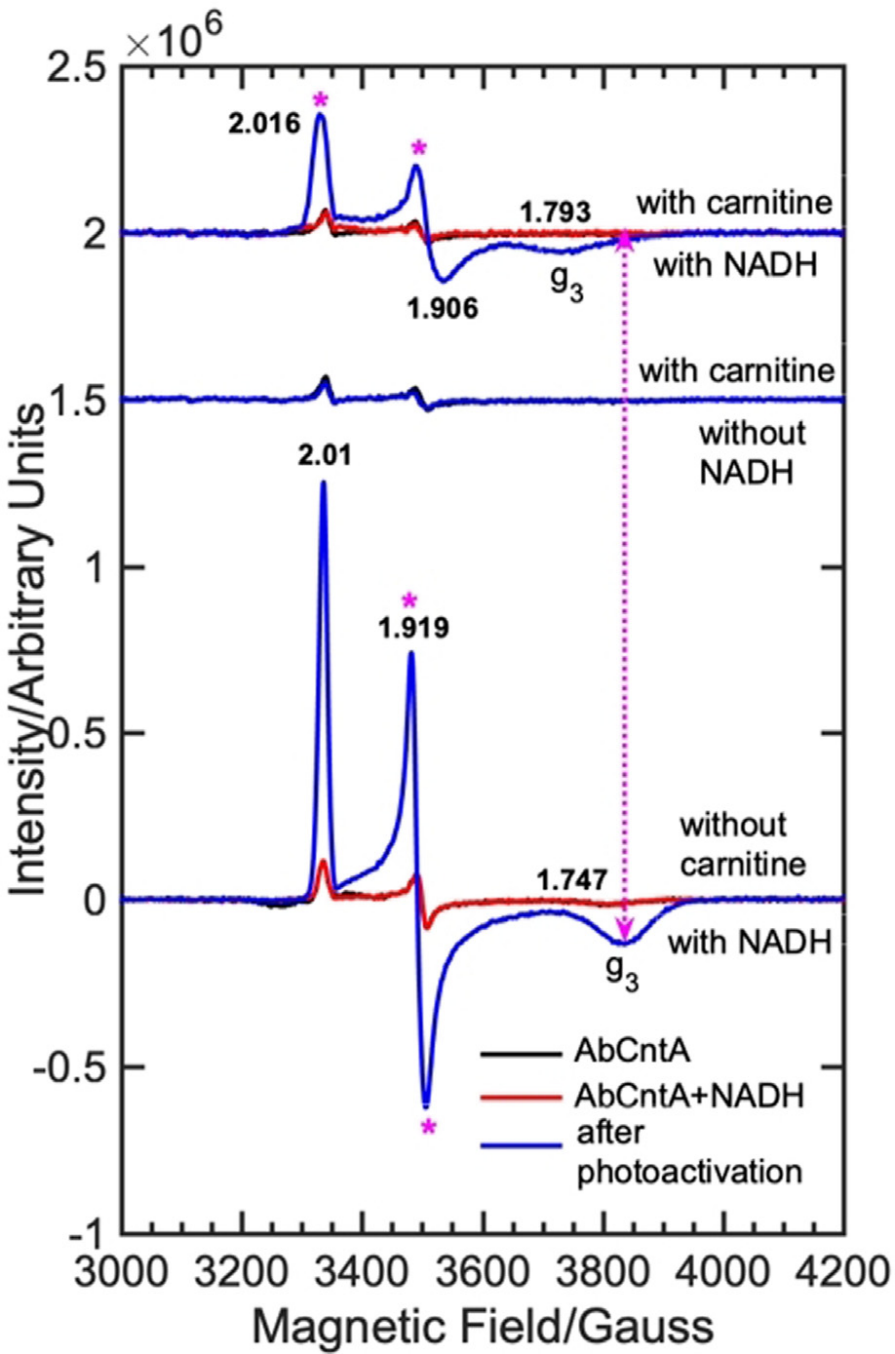
cw EPR spectra of the as‐isolated “*Ab*CntA‐WT” in the presence and absence of carnitine/NADH following photoexcitation of NADH using blue‐light, 365 nm; (bottom) *Ab*CntA‐WT+NADH in the absence of carnitine; (middle) *Ab*CntA‐WT+carnitine in the absence of NADH; (top) *Ab*CntA‐WT+NADH in the presence of carnitine. The double header arrowed, dotted magenta line indicates that the *g*_3_ component of the Rieske‐type, [2Fe‐2S]^+1^ EPR signal in the *Ab*CntA‐WT has been shifted towards low magnetic field upon substrate, carnitine binding. The magenta asterisks show the significant EPR line broadening observed in the *Ab*CntA‐WT+NADH+carnitine sample. *Conditions*; as described in the experimental section in the SI.

In Figure S5A and S5B, temperature‐dependent changes in the EPR spectrum of the photoactivated “*Ab*CntA‐WT+carnitine+NADH” are presented. When the sample was annealed at the specified temperatures for a set period of time as indicated in Figure S5C and S5D, the intensity of the EPR signals observed between 3200–4000 G continuously decreased until the temperature reached 260 K (≈0.1–0.2 ratio), above which it becomes constant within experimental error. Simultaneously, the spectrum shows negligibly changes in intensity for the EPR signal at *g*=4.3 associated with the high‐spin (*S=*5/2), ferric species at low‐magnetic fields, 500–2000 G (Figure S5A and S5C). However, the intensity of the EPR signal at *g*=4.3 grows significantly when the sample is annealed above 260 K for a longer time, in contrast to that observed between 3200–4200 G.

To shed light on the origin of the population of EPR signal at *g*=4.3 in “*Ab*CntA‐WT+carnitine+NADH” during annealing, similar experiments were performed on two control samples; (i) *Ab*CntA‐WT+NADH (ii) *Ab*CntA‐WT+carnitine. In contrast to that observed for “*Ab*CntA‐WT+carnitine+NADH”, no formation of the EPR signal at *g*=4.3 is observed when the “*Ab*CntA+carnitine” sample was annealed at 270 K for 20 min (Figure S6). However, it is noted that the residual EPR signal observed at *g*=4.3 before the annealing process continuously decreased following annealing of the sample at higher temperatures. It is often observed for a non‐heme iron center that autooxidation and/or degradation of the monoFe can lead to the formation of the high‐spin, ferric EPR signals at low magnetic field.[^1^, ^2^] If autooxidation and degradation of the *Ab*CntA‐WT were responsible for the observation of high‐spin, ferric EPR signal at *g*=4.3, a similar high‐spin, EPR signal would have been observed in *Ab*CntA‐WT+carnitine. However, this is not the case, suggesting that the EPR signal observed at *g*=4.3 in “*Ab*CntA+carnitine+NADH” is plausibly associated with the substrate turnover process.

Following photoactivation and annealing studies of “*Ab*CntA‐WT+carnitine”, identical experiments were performed on the frozen solution of “*Ab*CntA‐WT+NADH” at 20 K. In contrast to the “*Ab*CntA‐WT+carnitine”, intense EPR signals arise from the one‐electron reduced Rieske center are observed following photoactivation at 240 K. The intensity of these EPR signals decreased slowly when annealing at higher temperatures and ≈80–90 % (Figure S7) signals remain when “*Ab*CntA‐WT+NADH” was annealed for 20 min at 270 K compared to the “*Ab*CntA‐WT+carnitine+NADH”. This demonstrates that the reactivity of *Ab*CntA‐WT is altered upon carnitine binding at the active‐site, as reported for other non‐heme enzymes.[^12^, ^13^, ^22^] The substrate binding might have influenced the redox‐potential of the monoFe and thus facilitated the electron transfer from the reduced Rieske center to the monoFe and triggered the activation of molecular oxygen for substrate catalysis. This can account for the decay of the EPR signals arising from the reduced Rieske center. Similar substrate assisted/induced electron transfer and metallo‐reduction have been observed previously in the 2‐domain, copper containing nitrite reductases and several heme enzymes.[^16^, ^17^, ^20^, ^23^] It is noteworthy that the substrate (carnitine) turnover catalyzed by *Ab*CntA‐WT in the presence of the reductase domain, “*Ab*CntB+NADH” is vastly different from that by photoactivated NADH (Figure 3). At the conclusion of the reaction, the EPR spectrum of *Ab*CntB+NADH+*Ab*CntA‐WT+carnitine shows accumulation of two *S*=1/2 EPR signals (Figure 3; cyan trace) in the presence of a large excess of NADH (75 mM). These two EPR signals are severely overlapped; (i) one of the signals is due to the Rieske, [2Fe‐2S]^1+^ and/or EPR active signal arising from the monoFe (ii) given the large anisotropy often associated with the low‐spin, *S*=1/2 ferric state,[^23^, ^28^, ^29^] the intense, isotropic EPR signal observed at 3350 G could derive from the substrate with *g*_iso_=2.003, which requires additional experiments to probe this further. However, no accumulation of EPR signals are observed when an equimolar amount or a slight excess of NADH was used to drive the carnitine oxidation. The spectrum shows no evidence for the formation of high‐spin, *S=*5/2 ferric EPR signal at low magnetic field (Figure 3; cyan trace).

**Figure 3:**
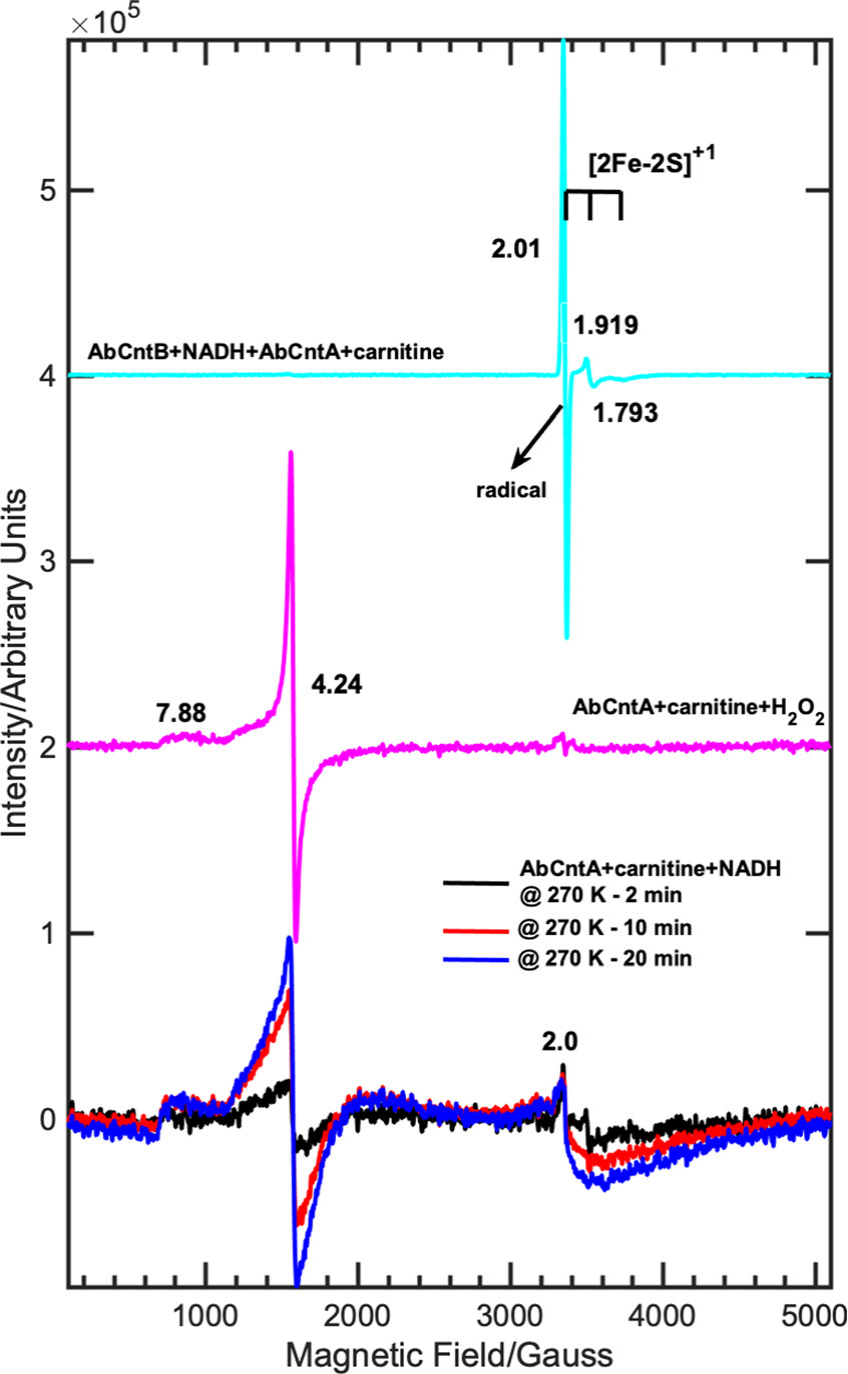
cw EPR spectra of the “*Ab*CntA‐WT” under various experimental conditions measured as a frozen solution at 20 K; (bottom traces) *Ab*CntA‐WT+carnitine+NADH following photoactivation and annealing at 270 K for the specified time in the legend; (middle; magenta trace) EPR spectrum of *Ab*CntA‐WT+carnitine+H_2_O_2_ shows the formation of high‐spin, *S*=5/2 ferric EPR signal; (top; cyan trace); EPR spectrum of *Ab*CntB+NADH+*Ab*CntA‐WT+carnitine shows the EPR signals arising from the *S*=1/2 species. The intensities of the magenta and cyan traces were manipulated to compare with the EPR spectra of the photoactivated *Ab*CntA‐WT+carnitine+NADH; *Conditions*; as described in the experimental section in the SI.

This is in contrast to the observations previously reported for other multi‐component, non‐heme monooxygenases, where high‐spin, ferric EPR signals were observed at the end of the catalytic cycle when reduced, oxygenases were reacted directly with the substrates.[^12^, ^13^] In addition, no radical EPR signal has been observed previously either alone or along with low/high‐spin ferric state for the multi‐component non‐heme enzymes. Interestingly, the annealing of the photoactivated “*Ab*CntA‐WT+carnitine+NADH” at 270 K (Figure 3; bottom—black, blue and red traces) leads to the formation of high‐spin, *S=*5/2 ferric EPR signal, which grows in intensity when annealed for longer times. The spectrum also shows EPR signals between 3000–4500 G, plausibly arising from a second EPR active species, *S*=1/2 rather than part of the high‐spin, *S=*5/2 EPR signal. The results from these two approaches strongly suggest that the photoactivated “*Ab*CntA‐WT+carnitine+NADH” follows a different reaction mechanism for the catalytic conversion of carnitine into TMA and MSA. However, these data do not provide details into the mechanisms and/or origin of the EPR signals, which were investigated further.

It has been reported that multi‐component, non‐heme enzymes, similar to that of P450 peroxygenases, react with hydrogen peroxide to turn over the specific substrates into products by the peroxide‐shunt mechanism with the formation of high‐spin, *S=*5/2 ferric EPR signals.[^12^, ^13^] To investigate such a mechanism, *Ab*CntA‐WT was reacted with hydrogen peroxide in the presence and absence of carnitine, respectively. As shown in Figures 3 (magenta trace) and S8 (red trace), the addition of H_2_O_2_ to “*Ab*CntA‐WT+carnitine” leads to the rapid formation of an EPR signal with *g*=4.3, arising likely from the ferric species with rhombic g tensor. The spectrum shows no evidence for the generation of one‐electron reduced Rieske center EPR signals at low magnetic fields between 3000–4500 G. The EPR spectrum recorded under identical experimental conditions without carnitine shows a negligibly small population of high‐spin, ferric EPR signals (Figure S8; black trace). Similar EPR signals have been reported when multi‐component naphthalene dioxygenase (NDO) was reacted with H_2_O_2_ in the presence and absence of naphthalene, where the rate of formation of the high‐spin EPR signal in NDO was correlated to the rate of formation of the dihydroxylated product, naphthalene *cis*‐diol, respectively.[^12^, ^13^] The comparisons of the EPR spectra of the photoactivated “*Ab*CntA‐WT+carnitine+NADH” at 270 K (Figure 3; bottom—black, blue and red traces) and H_2_O_2_ reacted “*Ab*CntA‐WT+carnitine” (Figure 3; middle—magenta trace) show similar high‐spin, *S=*5/2 ferric EPR signals in both cases. These observations suggest that the photoactivated “*Ab*CntA‐WT+carnitine+NADH” plausibly turns over carnitine into TMA and MSA, resulting in the formation of high‐spin, ferric EPR signals.

Earlier studies reported that photoexcitation of NADH using blue light, 355 nm injects an electron to the primary redox center in the copper containing nitrite reductase enzymes.[^16^, ^17^] This often results in rapid, one‐electron reduction followed by subsequent electron transfer process to initiate the catalytic reaction. Assuming that a similar process occurs in the photoactivated, “*Ab*CntA‐WT+carnitine+NADH” for the carnitine turnover, then one electron reduced (by dithionite) *Ab*CntA‐WT in the presence of carnitine would show a similar EPR spectrum to that of photoactivated “*Ab*CntA‐WT+carnitine+NADH” annealed at 270 K. Thus, the dithionite addition (RT) to *Ab*CntA‐WT in the presence (Figures S9,S10; red traces) and absence of carnitine (Figures S9 and S10; black traces) generated a one‐electron reduced Rieske center EPR signals, but no evidence for the formation of high‐spin, *S*=5/2 ferric EPR signals (Figure S8). It is noteworthy that higher concentration of buffer solution (see experimental section in SI) is used to compensate the effect of dithionite on the *p*H of the protein solution. However, comparison of the EPR spectra of photoactivated “*Ab*CntA‐WT+carnitine+NADH” at 240 K to that of dithionite reduced, “*Ab*CntA‐WT+carnitine” shows formation of an identical EPR species in both samples (Figures S11). Interestingly, the annealing at higher temperatures leads to the generation of high‐spin, *S*=5/2, ferric EPR signal and substrate turnover. Figure 4 illustrates our current hypothesis to account for the generation of high‐spin, *S*=5/2, ferric EPR signal and thus carnitine turnover. In the absence of carnitine (Figure 4 A), one‐electron reduced Rieske center EPR signals are formed following photoactivation of NADH with no further reaction, except slow decay of the reduced Rieske center EPR signals. However, in the presence of carnitine (Figure 4 B), electron transfer from the reduced Rieske center to the monoFe is triggered. This activates the molecular oxygen, resulting in the formation of one of three possible intermediates (X), followed by a high‐valent Fe(IV) and/or Fe(V)‐oxo species (not included in Figure 4), which reacts with carnitine to complete the oxidation reaction. The decay of the reduced Rieske center and formation of high‐spin, *S*=5/2 ferric centers satisfying the two single‐electron ([2Fe‐2S]^1+^ to [2Fe‐2S]^2+^ and Fe^2+^/Fe^3+^) chemistry required for carnitine conversion into TMA and MSA, as observed in other non‐heme enzymes.[^12^, ^13^]

**Figure 4:**
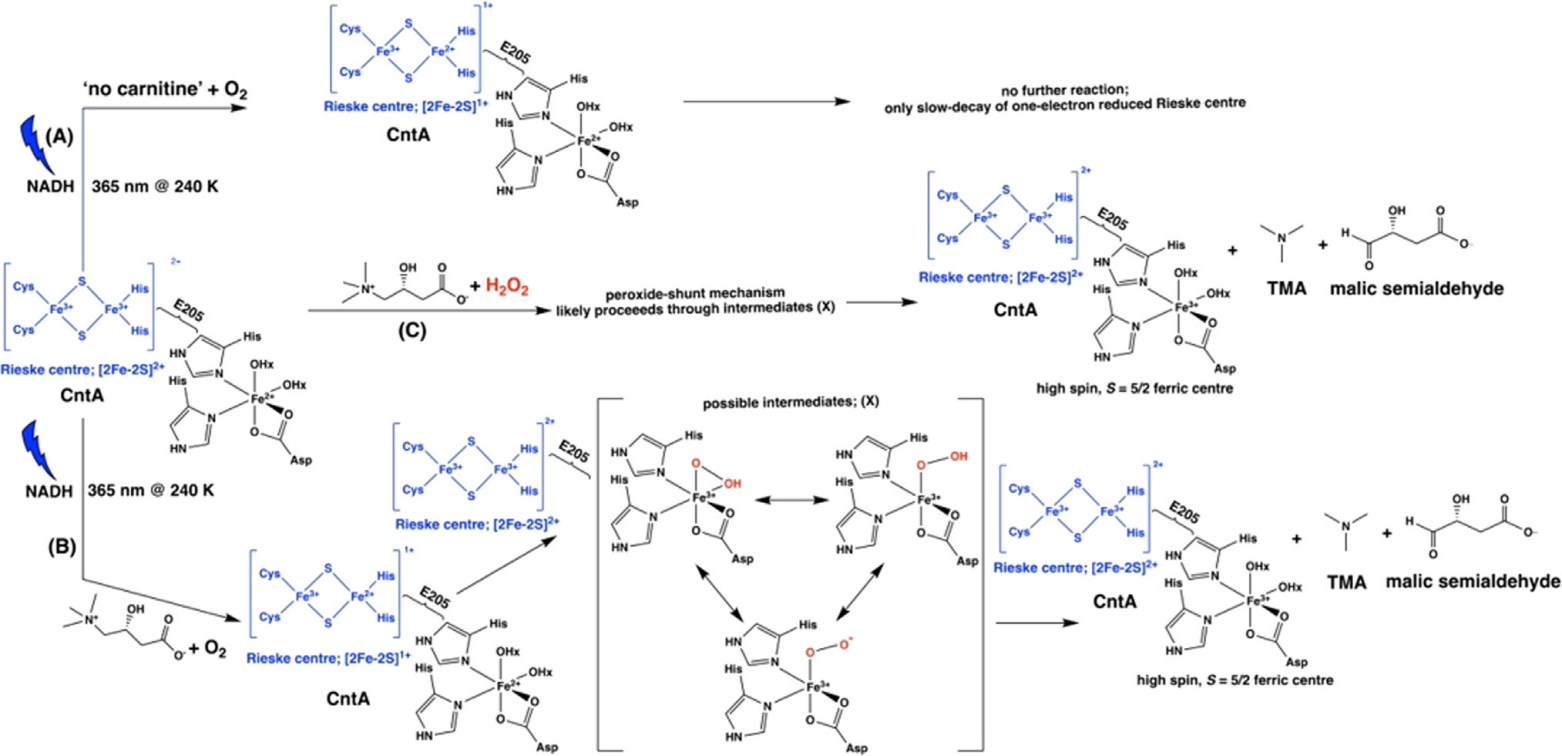
Proposed reaction Scheme for the oxidation reaction of carnitine catalyzed by *Ab*CntA‐WT in the presence of photoactivated NADH; *Ab*CntA+NADH photoactivation in the absence (A) and presence (B) of carnitine; (C) peroxide‐shunt reaction. The coordinated oxygenic ligands around the ferric center are only for an illustrative purpose, but other possibilities cannot be ruled out.

The following two scenarios cannot be ruled out; (i) under the experimental conditions used here, H_2_O_2_ might have been produced plausibly from the oxidized NADH, which subsequently reacted with “*Ab*CntA‐WT+carnitine” via the peroxide‐shunt mechanism (Figure 4 C) to generate the high‐spin, *S=*5/2 ferric EPR signal. Formation of H_2_O_2_ from the oxidized NADH has been reported previously;[^24^, ^25^] (ii) Formation of H_2_O_2_ due to an uncoupling reaction, has been observed for other heme and non‐heme enzymes.[^22^, ^26^, ^27^] The EPR signals observed between 3000–4500 G is likely due to an equilibrium between low‐spin (*S*=1/2 ) and high‐spin (*S*=5/2) ferric conformation as observed in cytochrome P450‐CYP121.^[30]^ To determine the exact nature of the reactive species responsible for the catalytic activity and unambiguous assignments requires advanced pulsed‐EPR studies and is currently under progress.

Earlier biochemical studies showed that the mutation of the bridging glutamate into alanine, E205A led to significant loss of the *Ab*CntA catalytic activity.^[9]^ To understand the role of E205, NADH‐photoactivation studies were performed on the *Ab*CntA‐E205A+NADH without substrate (Figures S13–S15). The time‐dependent NADH‐photoactivation leads to the formation of one‐electron reduced Rieske center in *Ab*CntA‐E205A+NADH (Figure S13), however significantly less population compared to that of *Ab*CntA‐WT+NADH (Figure S15). When annealing was performed at higher temperatures (above 240 K) for the *Ab*CntA‐E205A+NADH, the EPR signals arise from the reduced Rieske center slowly decayed (Figure S14; top) and only ≈30 % signal remains at 275 K (Figure S14; bottom) in contrast to the *Ab*CntA‐WT+NADH, where ≈80–90 % of signal remains in the reduced state (Figure S7; C). These observations demonstrate that the significantly reduced catalytic activity associated with the *Ab*CntA‐E205A is plausibly due to the disruption in the electron transfer process and its incompetence to stabilize the one‐electron reduced Rieske center to carryout multiple turnovers.

In summary, the photoactivation of NADH combined with the EPR spectroscopic method can be used as a new‐spectroscopic tool to track down the electron transfer process in the multi‐component, non‐heme iron proteins. Our approach highlights the possibilities of trapping and characterizing the elusive intermediates at low temperature without interference from other EPR active signals, as in the case of the ^60^Co‐cryolitic reduction method (strong organic radicals observed with g ≈2.001–2.002), thus opening up the possibilities to characterizing redox centers with g tensor closer to free electron value. It allows the direct monitoring of electron transfer process, decay and formation of EPR active signals simultaneously. The photoactivation studies performed on the *Ab*CntA‐E205A mutant demonstrate that interruption in the electron transfer process and the loss of stability in the one‐electron reduced, [2Fe‐2S]^1+^ might be associated with the significant loss in catalytic activity in the *Ab*CntA‐E205A mutant. In some cases, the solvated electrons and solvent radicals might cause damage to the protein structure/activity, however, the control experiments confirm this is not the case in our study when NADH is photoactivated. This new approach is general and could be used to trap the elusive intermediates to better understand the catalytic mechanisms in other multi‐component, non‐heme enzymes,^[31]^ copper‐containing enzymes (lytic polysaccharide monooxygenase; LPMO)^[32]^ and non‐heme diiron centers like ribonucleotide reductase (RNR),^[33]^ methane monooxygenase (MMOs),^[34]^ aldehyde‐deformylating oxygenases (ADO)^[35]^ respectively.

## Conflict of interest

The authors declare no conflict of interest.

## Supporting information

As a service to our authors and readers, this journal provides supporting information supplied by the authors. Such materials are peer reviewed and may be re‐organized for online delivery, but are not copy‐edited or typeset. Technical support issues arising from supporting information (other than missing files) should be addressed to the authors.

Supplementary

## References

[1] P. C. A. Bruijnincx , G. V. Koten , R. J. M. K. Gebbink , Chem. Soc. Rev. 2008, 37, 2716–2744. 19020684 10.1039/b707179p

[2] E. I. Solomon , T. C. Brunold , M. I. Davis , J. N. Kemsley , S.-K. Lee , N. Lehnert , F. Neese , A. J. Skulan , Y.-S. Yang , J. Zhou , Chem. Rev. 2000, 100, 235–349. 11749238 10.1021/cr9900275

[3] E. G. Kovaleva , M. B. Neibergall , S. Chakrabarty , J. D. Lipscomb , Acc. Chem. Res. 2007, 40, 475–483. 17567087 10.1021/ar700052v PMC2720168

[4] S. H. Zeisel , M. Warrier , Annu. Rev. Nutr. 2017, 37, 157–181. 28715991 10.1146/annurev-nutr-071816-064732

[5] W. H. Tang , S. L. Hazen , Circulation 2017, 135, 1008–1010. 28289004 10.1161/CIRCULATIONAHA.116.024251 PMC5354081

[6] Z. Jie , et al., Nat. Commun. 2017, 8, 845. 29018189 10.1038/s41467-017-00900-1 PMC5635030

[7] S. Craciun , E. P. Balkus , Proc. Natl. Acad. Sci. USA 2012, 109, 21307–21312. 23151509 10.1073/pnas.1215689109 PMC3535645

[8] G. Kalnins , J. Kuka , S. Gringerga , M. Makrecka-Kuka , E. Liepinsh , M. Dambrova , K. Tars , J. Biol. Chem. 2015, 290, 21732–21740. 26187464 10.1074/jbc.M115.670471 PMC4571895

[9a] Y. Zhu , E. Jameson , M. Crosatti , H. Schafer , K. Rajakumar , T. D. H. Bugg , Y. Chen , Proc. Natl. Acad. Sci. USA 2014, 111, 4268–4273; 24591617 10.1073/pnas.1316569111 PMC3964110

[9b] M. Quareshy , M. Shanmugam , E. Townsend , E. Jameson , T. D. H. Bugg , A. D. Cameron , Y. Chen , J. Biol. Chem. 2020, 10.1074/jbc.RA120.016019. PMC7948474 33158989

[10] K. D. Daughtry , Y. Xiao , D. Stoner-Ma , E. Cho , A. M. Orville , P. Liu , K. N. Allen , J. Am. Chem. Soc. 2012, 134, 2823–2834. 22224443 10.1021/ja2111898 PMC5718839

[11a] A. Karlsson , J. V. Parales , R. E. Parales , D. T. Gibson , H. Eklund , S. Ramaswamy , Science 2003, 299, 1039–1042; 12586937 10.1126/science.1078020

[11b] R. L. D'Ordine , T. J. Rydel , M. J. Storek , E. J. Sturman , F. Moshiri , R. K. Bartlett , G. R. Brown , R. J. Eilers , C. Dart , Y. Qi , S. Flasinski , S. J. Franklin , J. Mol. Biol. 2009, 392, 481–497. 19616009 10.1016/j.jmb.2009.07.022

[12] M. D. Wolfe , J. D. Lipscomb , J. Biol. Chem. 2003, 278, 829–835. 12403773 10.1074/jbc.M209604200

[13] M. D. Wolfe , J. V. Parales , D. T. Gibson , J. D. Lipscomb , J. Biol. Chem. 2001, 276, 1945–1953. 11056161 10.1074/jbc.M007795200

[14] Y. Orii , Biochemistry 1993, 32, 11910–11914. 8218263 10.1021/bi00095a021

[15] Y. Orii , T. Miki , K. Kakinuma , Photochem. Photobiol. 1995, 61, 261–268. 7716188 10.1111/j.1751-1097.1995.tb03969.x

[16] T. M. Hedison , R. T. Shenoy , A. I. Iorgu , D. J. Heyes , K. Fisher , G. S. A. Wright , S. Hay , R. R. Eady , S. V. Antonyuk , S. S. Hasnain , N. S. Scrutton , ACS Catal. 2019, 9, 6087–6099. 32051772 10.1021/acscatal.9b01266 PMC7007197

[17] S. Brenner , D. J. Heyes , S. Hay , M. A. Hough , R. R. Eady , S. S. Hasnain , N. S. Scrutton , J. Biol. Chem. 2009, 284, 25973–25983. 19586913 10.1074/jbc.M109.012245 PMC2757998

[18] R. Davydov , T. M. Makris , V. Kofman , D. E. Werst , S. G. Sligar , B. M. Hoffman , J. Am. Chem. Soc. 2001, 123, 1403–1415. 11456714 10.1021/ja003583l

[19a] T. M. Hedison , M. Shanmugam , D. J. Heyes , R. Edge , N. S. Scrutton , Angew. Chem. Int. Ed. 2020, 59, 13936–13940; 10.1002/anie.202005052 PMC7497095 32352195

[20] R. Davydov , A. A. Gilep , N. V. Strushkevich , S. A. Usanov , B. M. Hoffman , J. Am. Chem. Soc. 2012, 134, 17149–17156. 23039857 10.1021/ja3067226 PMC3491644

[21] R. M. Davydov , M. P. McLaughlin , E. Bill , B. M. Hoffman , P. L. Holland , Inorg. Chem. 2013, 52, 7323–7325. 24004284 10.1021/ic4011339 PMC3767189

[22] P. Wende , F. H. Bernhardt , K. Pfleger , Eur. J. Biochem. 1989, 181, 189–197. 2714278 10.1111/j.1432-1033.1989.tb14710.x

[23] S. Matthews , J. D. Belcher , K. L. Tee , H. M. Girvan , K. J. McLean , S. E. J. Rigby , C. W. Levy , D. Leys , D. A. Parker , R. T. Blankley , A. W. Munro , J. Biol. Chem. 2017, 292, 5128–5143. 28053093 10.1074/jbc.M116.762336 PMC5377825

[24] T. Ramasarma , A. Swaroop , W. Mackellar , F. L. Crane , J. Bioenerg. Biomembr. 1981, 13, 241–252. 7334020 10.1007/BF00743203

[25] D. A. Webster , J. Biol. Chem. 1975, 250, 4955–4958. 238973

[26] K. Lee , J. Bacteriol. 1999, 181, 2719–2725. 10217759 10.1128/jb.181.9.2719-2725.1999 PMC93710

[27] G. D. Nordblom , M. J. Coon , Arch. Biochem. Biophys. 1977, 180, 343–347. 18091 10.1016/0003-9861(77)90047-9

[28] O. Y. Lyakin , K. P. Bryliakov , G. J. P. Britovsek , E. P. Talsi , J. Am. Chem. Soc. 2009, 131, 10798–10799. 19722657 10.1021/ja902659c

[29a] F. T. de Oliveira , A. Chanda , D. Banerjee , X. Shan , S. Mondal , L. Que, Jr. , E. L. Bominaar , E. Munck , T. J. Collins , Science 2007, 315, 835–838; 17185561 10.1126/science.1133417

[29b] C. Kim , K. Chen , J. Kim , L. Que, Jr. , J. Am. Chem. Soc. 1997, 119, 5964–5965.

[30] A. J. Fielding , K. Dornevil , L. Ma , I. Davis , A. Liu , J. Am. Chem. Soc. 2017, 139, 17484–17499. 29090577 10.1021/jacs.7b08911 PMC5765751

[31] P. L. Herman , M. Behrens , S. Chakraborty , B. M. Chrastil , J. Barycki , D. P. Weeks , J. Biol. Chem. 2005, 280, 24759–24767. 15855162 10.1074/jbc.M500597200

[32] C. H. Kjaergaard , M. F. Qayyum , S. D. Wong , F. Xu , G. R. Hemsworth , D. J. Walton , N. A. Young , G. J. Davies , P. H. Walton , K. S. Johansen , K. O. Hodgson , B. Hedman , E. I. Solomon , Proc. Natl. Acad. Sci. USA 2014, 111, 8797–8802. 24889637 10.1073/pnas.1408115111 PMC4066490

[33] M. Shanmugam , P. E. Doan , N. S. Lees , J. Stubbe , B. M. Hoffman , J. Am. Chem. Soc. 2009, 131, 3370–3376. 19220056 10.1021/ja809223s PMC2789976

[34] V. J. DeRose , K. E. Liu , S. J. Lippard , B. M. Hoffman , J. Am. Chem. Soc. 1996, 118, 121–134.

[35] L. J. Rajakovich , H. Norgaard , D. M. Warui , W. Chang , N. Li , S. J. Booker , C. Krebs , J. M. Bollinger, Jr. , M. E. Pandelia , J. Am. Chem. Soc. 2015, 137, 11695–11709. 26284355 10.1021/jacs.5b06345

[36] M. Massmig , E. Reijerse , J. Krausze , C. Laurich , W. Lubitz , D. Jahn , J. Moser , J. Biol. Chem. 2020, 295, 13065–13078. 32694223 10.1074/jbc.RA120.014266 PMC7489909

