# Supplementary material for: Light‐Activated Electron Transfer and Catalytic Mechanism of Carnitine Oxidation by Rieske‐Type Oxygenase from Human Microbiota
Source: Angew Chem Int Ed Engl. 2020 Dec 28;60(9):4529–34. doi: 10.1002/anie.202012381 (PMC7986066; doi:10.1002/anie.202012381)
Supplement: Supplementary file 1 — Supplementary [file ANIE-60-4529-s001.pdf]

Angewandte Chemie
Eine Zeitschrift der Gesellschaft Deutscher Chemiker

Supporting Information

Light-Activated Electron Transfer and Catalytic Mechanism of Carnitine Oxidation by Rieske-Type Oxygenase from Human Microbiota

Muralidharan Shanmugam, Mussa Quareshy, Alexander D. Cameron, Timothy D. H. Bugg, and Yin Chen*

anie_202012381_sm_miscellaneous_information.pdf

EXPERIMENTAL METHODS

All reagents were of analytic grade and were purchased from Sigma-Aldrich (Dorset, UK). The Rieske-type, $[2Fe\text{-}2S]^{2+}$ cluster and catalytic, mononuclear non-heme iron containing *acinetobacter baumannii (Ab), Ab*CntA oxygenase and *Ab*CntA NADH reductase domains were independently expressed and purified using previously published protocols.[1] All EPR samples were prepared in a 10 mM HPEPS buffer with 250 mM NaCl, 0.5mM TCEP and 10 % glycerol (v/v) (pH 7.6) in an aerobic condition. Samples containing *approx.* 200 μM *Ab*CntA, 75 mM nicotinamide adenine dinucleotide (NADH) were transferred into 4 mm Suprasil quartz EPR tubes (Wilmad LabGlass) and frozen in liquid N_2. The photoactivation of NADH was carried out at 240 K by placing the *Ab*CntA-WT sample (in the presence/absence of carnitine) in a 1-propanol and liquid nitrogen solvent mixture. The direct illumination of the sample (for the specified duration described in the text) was performed using Thorlabs M365L2 mounted LED[2], which has a nominal wavelength of 365 nm. The output power of the LED was 360 mW. All EPR samples were measured on a Bruker ELEXSYS-E580 X-band EPR spectrometer with the microwave power set to 30 dB (0.2 mW), the modulation amplitude set to 5 G, a time constant of 41 ms, a conversion time of 41 ms, a sweep time of 84 s, the receiver gain set to 60 dB and an average microwave frequency of 9.384 GHz. All annealing measurements were performed using a 1-propanol and liquid nitrogen solvent mixture, and all samples were annealed for the specified times (see main text) at the temperature stated. All EPR spectra were measured as a frozen solution at 20 K. The analysis of the continuous wave EPR spectra and simulations were performed using EasySpin toolbox (5.2.28) for the Matlab program package.[3] The extracted spin-Hamiltonian parameters for the one-electron reduced, Rieske $[2Fe\text{-}2S]^{1+}$ are similar to the previously reported values.[4] It is noteworthy, like the majority of other cryotrapping-EPR approaches,[5] NADH photoactivation at 240 K is semiquantitative, and no firm thermodynamic parameters were derived from these measurements. Similar to the reported ^{60}Co-γ-irradiation studies on the metalloenzymes,[6,7] this approach is used to identify the electron transfer process and active intermediate states produced during the catalytic cycle of these enzymes.

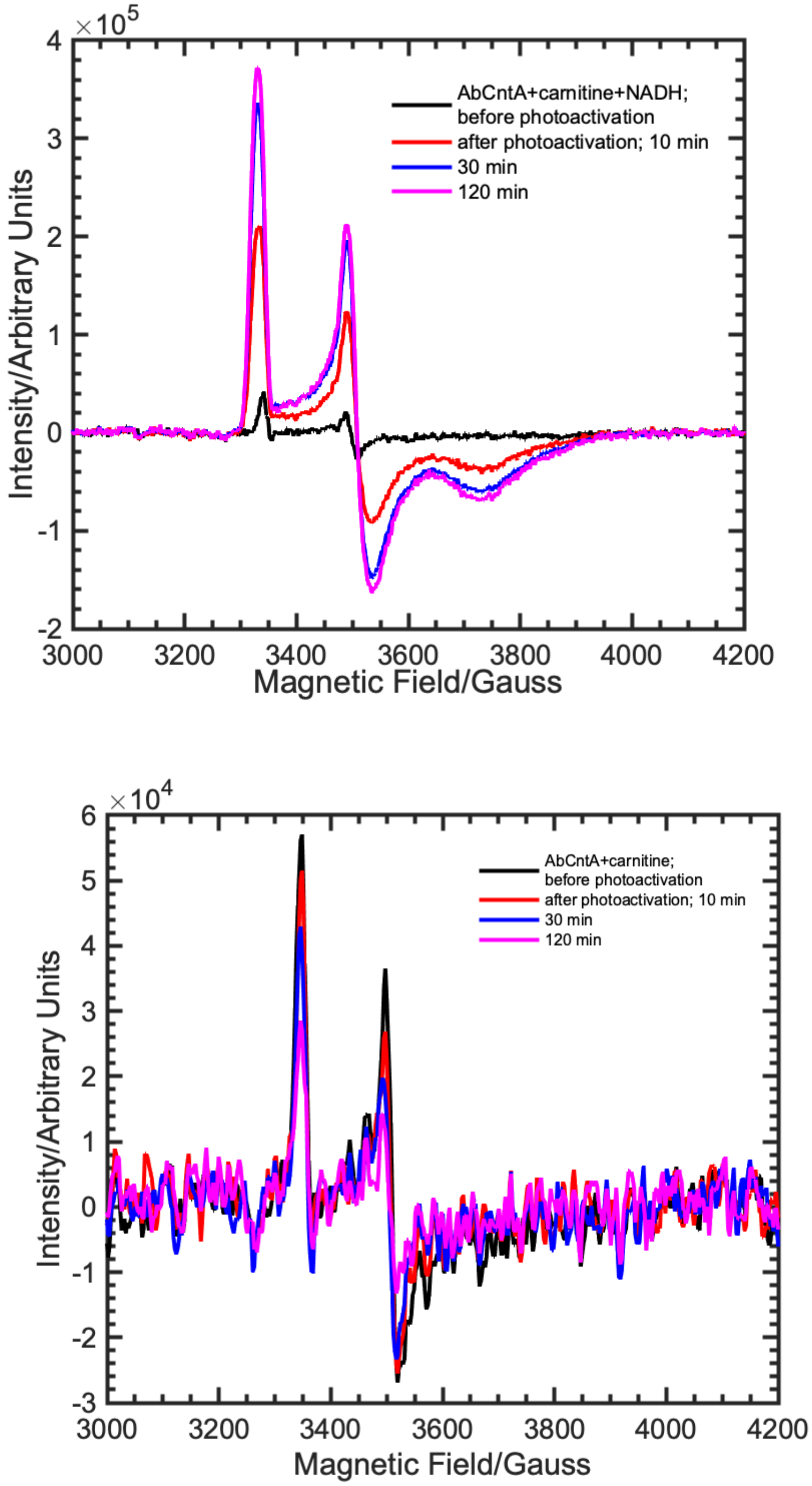

Figure S1. cw-EPR spectra of the '*Ab*CntA+carnitine' in the presence (top) and absence (bottom) of NADH, following photoactivation using blue-light, 365 nm for various time scale. The spectra were measured as a frozen solution at 20 K. *Conditions* – as described in the experimental section.

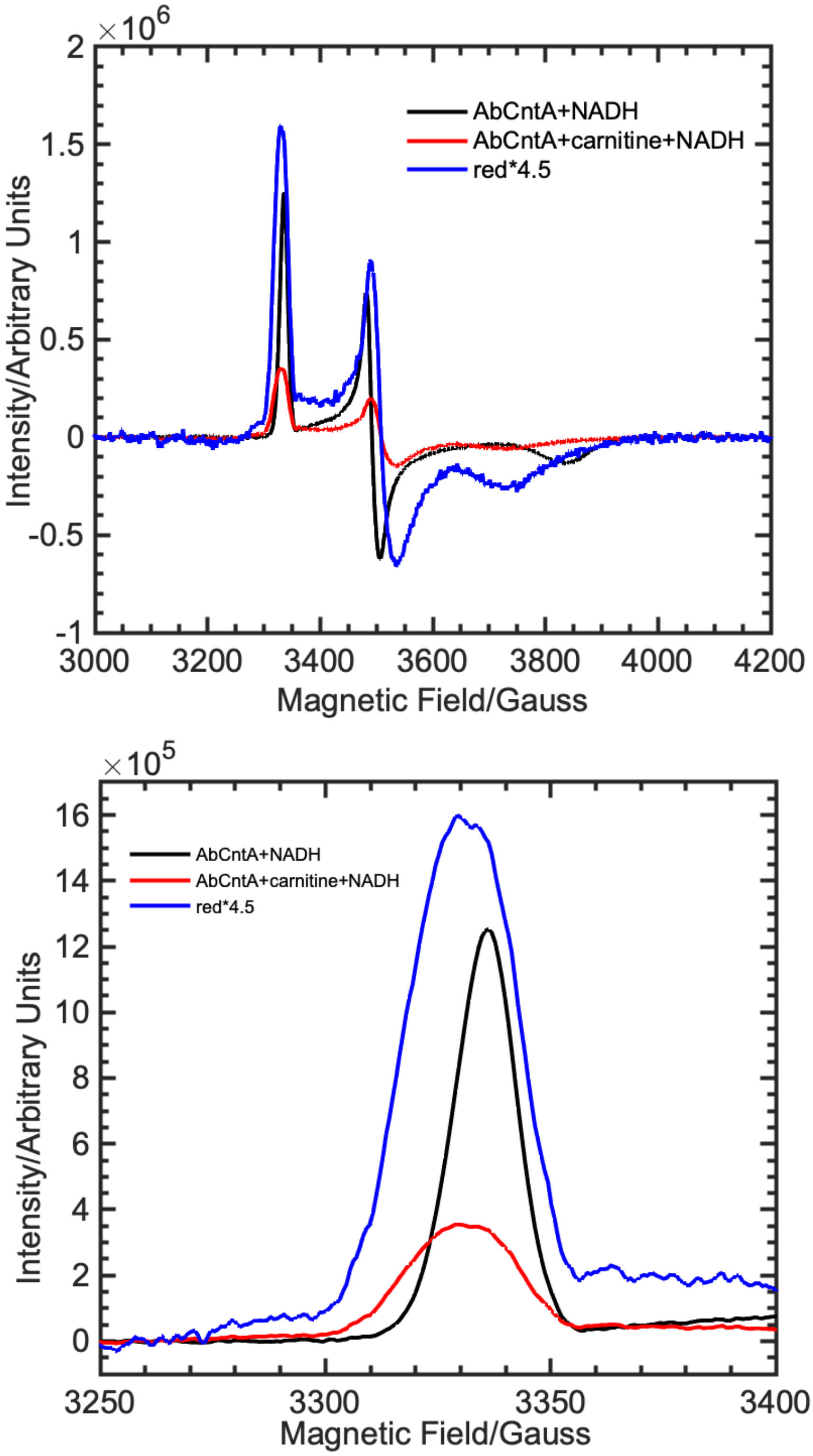

Figure S2. Comparisons of cw-EPR spectra of the '*Ab*CntA' in the presence (red and blue traces) and absence (black trace) of substrate, carnitine, following photoactivation using blue-light, 365 nm for 30 min. The EPR spectra are expanded between 3250-3400 G (bottom) to show the overlapping signals arising from two different EPR active species and the EPR line broadening. *Conditions* – as described in the experimental section.

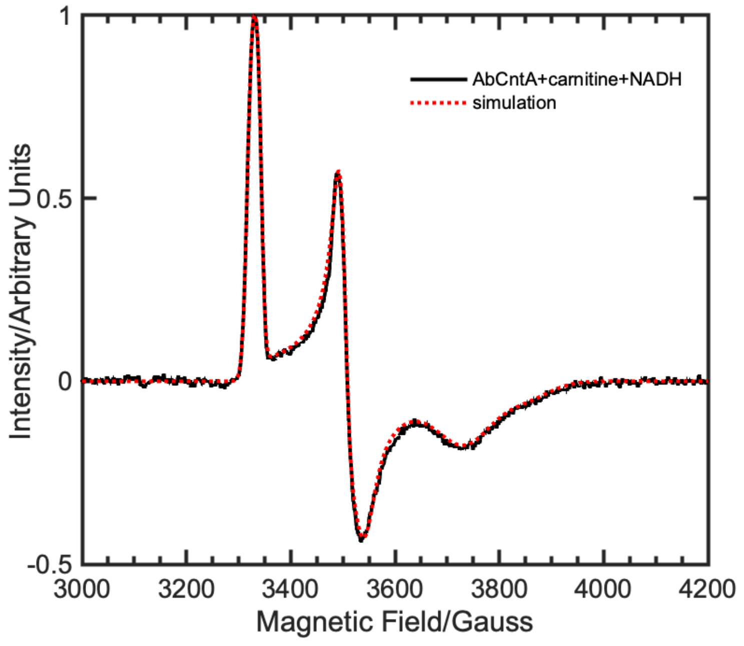

Figure S3. Experimental (black trace) and simulated (red dotted line) EPR spectra of the photoactivated '*Ab*CntA+carnitine+NADH' measured as a frozen solution at 20 K. The spectrum was successfully simulated by considering two contributing, $S = ½$ spin species with the following spin-Hamiltonian parameters (also provided in Table S1): species(1); ***g*** = [1.793 1.906 2.016], line widths = [0.12 0.1] mT; HStrain = [273 120 61] MHz; weight = 0.7; species(2); ***g*** = [1.741 1.914 2.008], line widths = [0.12 0.1] mT; HStrain = [273 54 42] MHz; weight = 0.3;

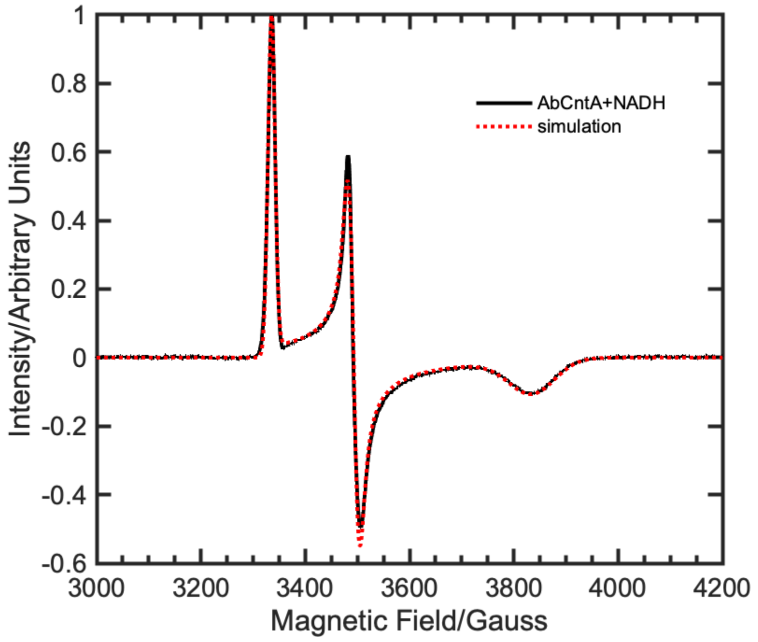

Figure S4. Experimental (black trace) and simulated (red dotted line) EPR spectra of the photoactivated '*Ab*CntA+NADH' measured as a frozen solution at 20 K. The spectrum was successfully with the following spin-Hamiltonian parameters (also provided in Table S1); ***g*** = [1.747 1.919 2.010], line widths = [0.12 0.1] mT; HStrain = [253 56 43] MHz;

Table S1. Spin-Hamiltonian Parameters used for the simulations of the EPR spectra of the one-electron reduced, Rieske-type, $[2Fe\text{-}2S]^{1+}$ in *Ab*CntA-WT.

Samples	EPR active signal	g-tensor	g_{iso}	Line widths[a] (mT)	H-strain[a] (MHz)
*Ab*CntA-WT+NADH	$[2Fe\text{-}2S]^{1+}$	[2.010 1.919 1.747]	1.892	[0.12 0.1]	[253 56 43]
*Ab*CntA-WT+carnitine+NADH	$[2Fe\text{-}2S]^{1+}$;[b]	[2.016 1.906 1.793]	1.905	[0.12 0.1]	[273 120 61]
	$[2Fe\text{-}2S]^{1+}$;[c]	[2.008 1.914 1.741]	1.888	[0.12 0.1]	[273 54 42]

[a] The lineshape of the spectra was reproduced by considering an isotropic Voigtian lineshape and an anisotropic broadening(H-Strain) respectively.

[b] weight = 70 %; [c] weight = 30 %

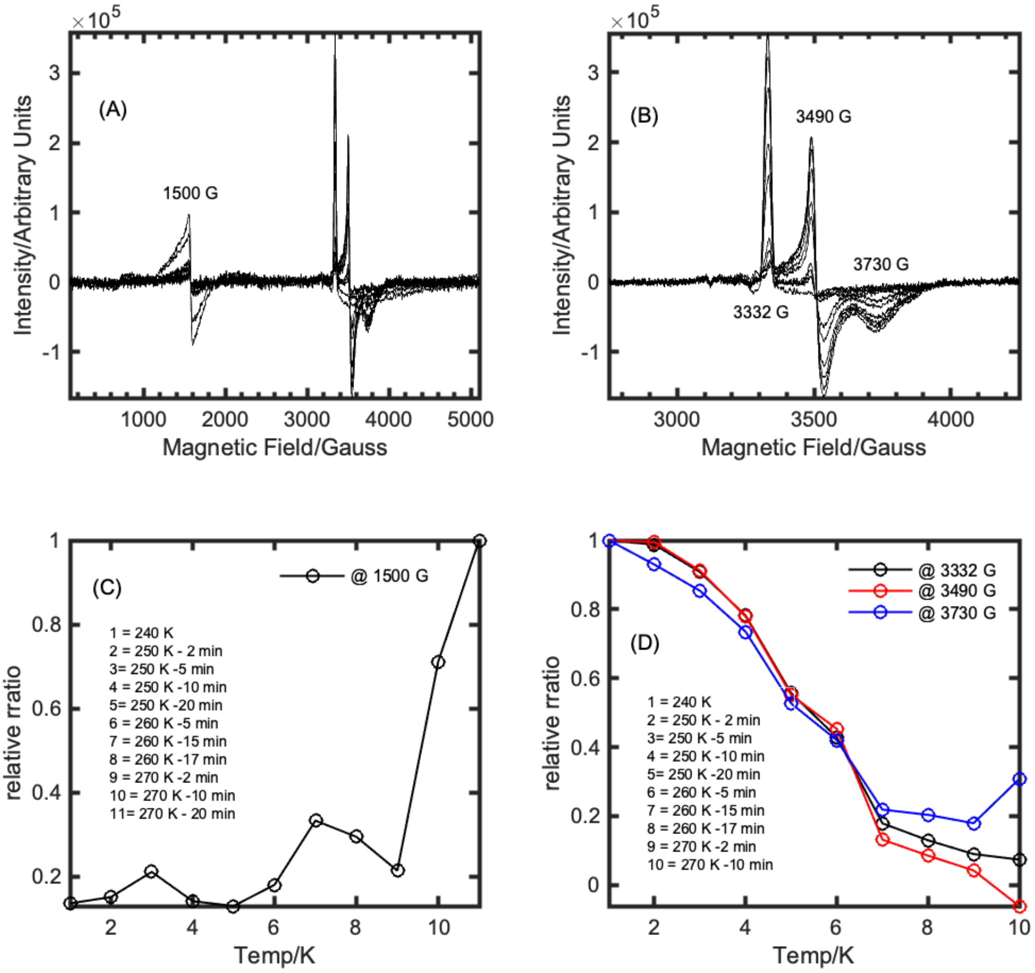

Figure S5. cw EPR spectra of the '*Ab*CntA-WT+carnitine+NADH' following photoactivation and annealing at the specified temperatures/time. (A,C) wide-sweep, cw-EPR spectra of '*Ab*CntA-WT+carnitine+NADH' shows the formation of high-spin (S=5/2), ferric EPR signal; (B,D) narrow-sweep, cw-EPR spectra of '*Ab*CntA-WT+carnitine+NADH' shows the decay of EPR signals arising from a S = ½ spin-state observed between 3200-4000 G; (C) The EPR spectra are normalised relative to the EPR signal observed at 270 K with an annealing time of 20 minutes to demonstrate the formation of the g = 4.3 EPR signals following annealing; (D) The EPR spectra are normalised relative to the EPR signal observed at 240 K before the annealing to demonstrate the decay of the EPR signals; All EPR spectra were measured as a frozen solution at 20 K. *Conditions;* as described in the experimental section.

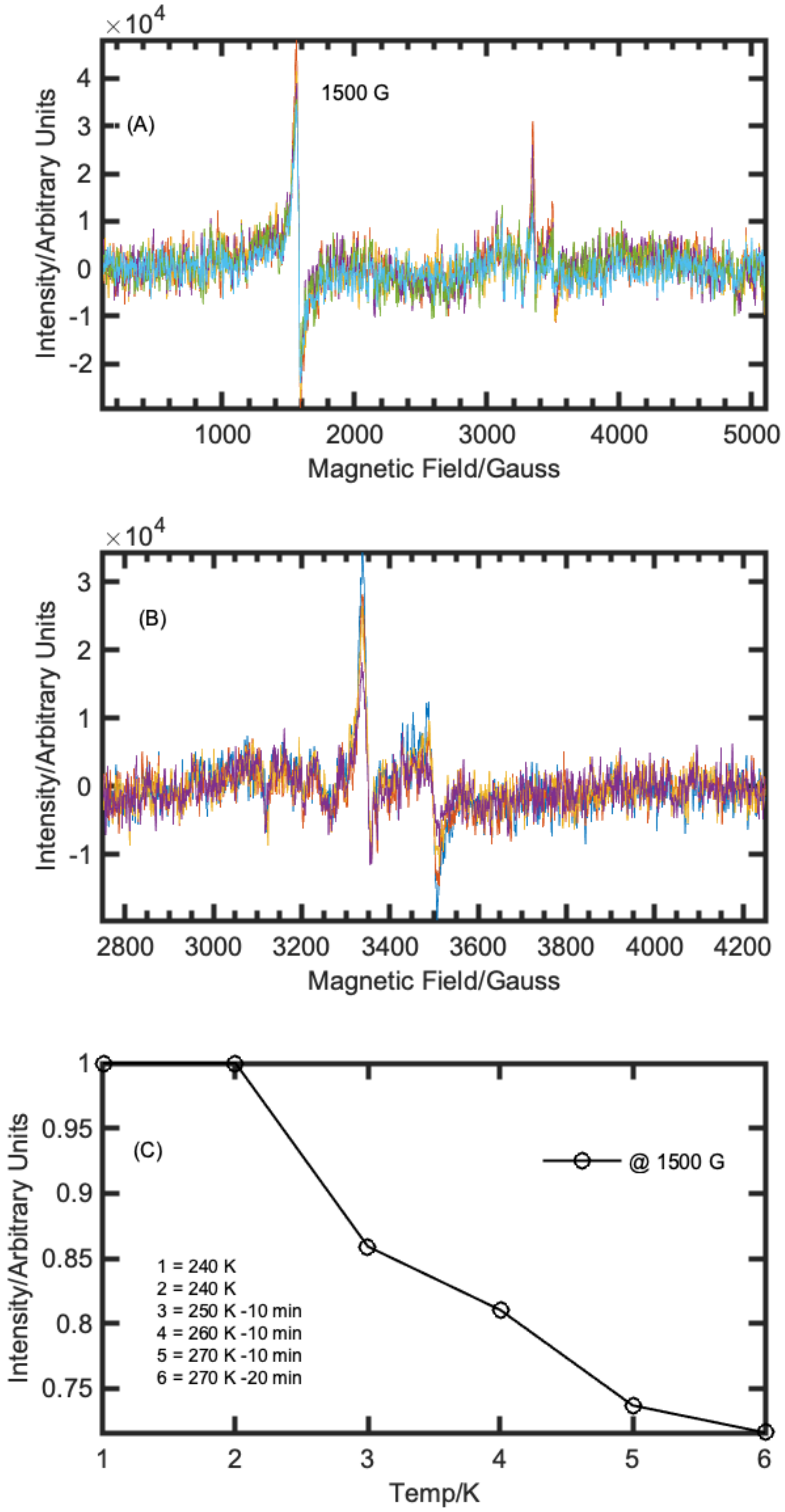

Figure S6. EPR spectral changes observed when "*Ab*CntA+carnitine" was annealed at the specified temperatures/times following photoexcitation using 365 nm, blue-light for 120 minutes. (A, C) wide-sweep, cw-EPR spectra of '*Ab*CntA+carnitine' monitoring the changes in intensity of the high-spin (*S*=5/2), ferric EPR signal when annealed at higher temperatures; (B) narrow-sweep, cw-EPR spectra of '*Ab*CntA+carnitine' to monitor the EPR signals arising from an *S* = ½ species;

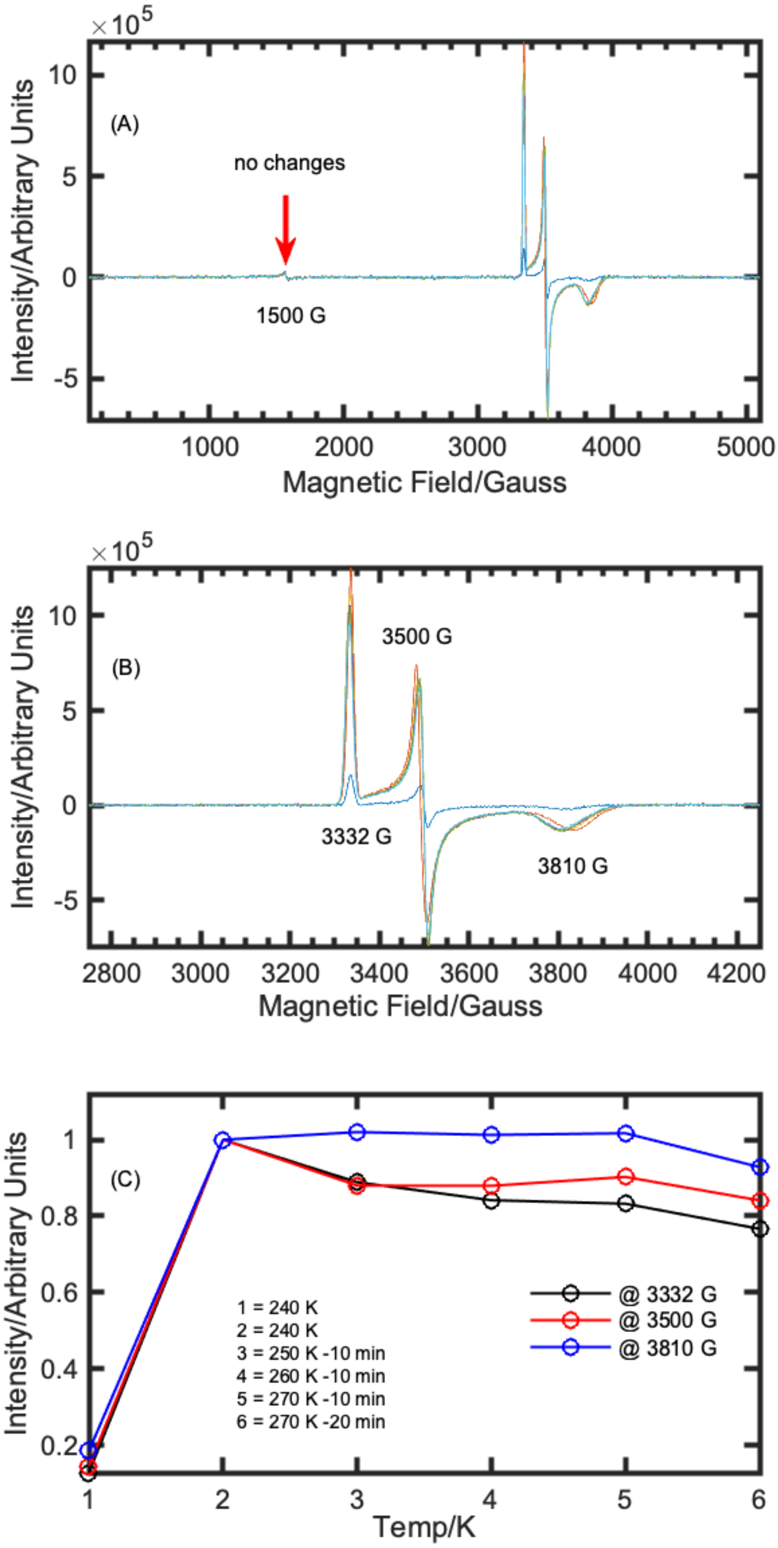

Figure S7. EPR spectral changes observed when "*Ab*CntA+NADH" was annealed at the specified temperatures/times, following photoexcitation using 365 nm, blue-light for 30 minutes. (A) wide-sweep, cw-EPR spectra of '*Ab*CntA+NADH' show no change in intensity of the EPR signals arising from a high spin, (*S*=5/2), ferric EPR signal when annealed at higher temperatures; (B,C) narrow-sweep, cw-EPR spectra of '*Ab*CntA+NADH' monitoring the changes in intensity of the one-electron reduced, Rieske [2Fe-2S]$^{+1}$ EPR signal when annealed at higher temperatures;

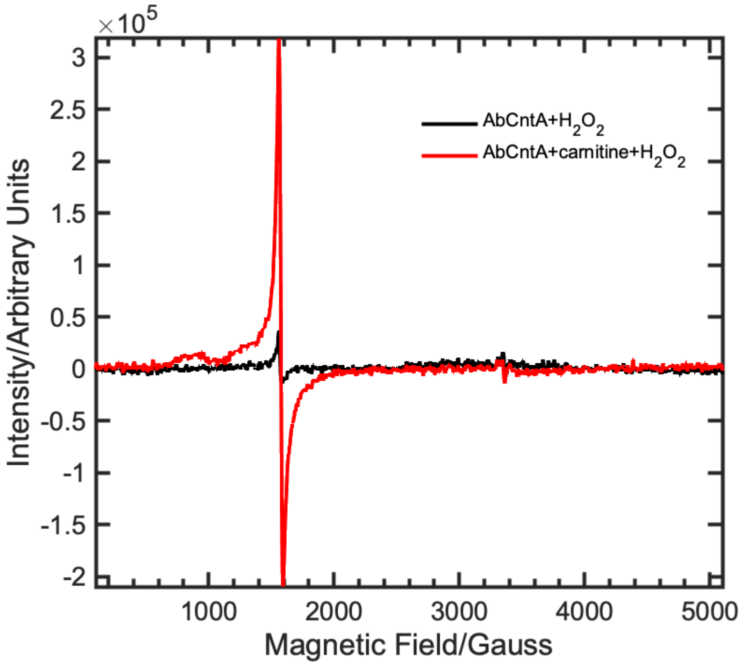

Figure S8. cw-EPR spectra of "as-isolated" *Ab*CntA+H_2O_2 in the presence (red trace) and absence (black trace) of the substrate, carnitine show the formation of high-spin, $S = 5/2$ ferric EPR signals. Both spectra have been background subtracted to remove the residual EPR signal arising from the empty EPR cavity. *Conditions* – as described in the experimental section.

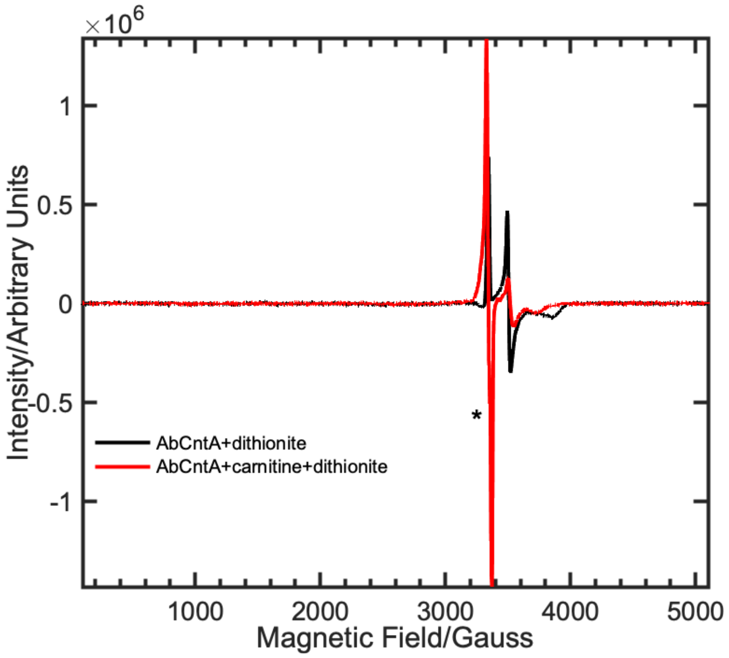

Figure S9. wide-sweep cw-EPR spectra of dithionite reduced, "as-isolated" *Ab*CntA in the presence (red trace) and absence (black trace) of the substrate, carnitine show the formation of one-electron reduced, Rieske, $[2Fe\text{-}2S]^{+1}$ EPR signals. There is no evidence for the generation of high-spin, $S = 5/2$ ferric EPR signals in the EPR spectra. The black asterisk mark indicates that this strong EPR signal arise from the quartz-impurity in the EPR tube (See Figure S10). *Conditions* – as described in the experimental section.

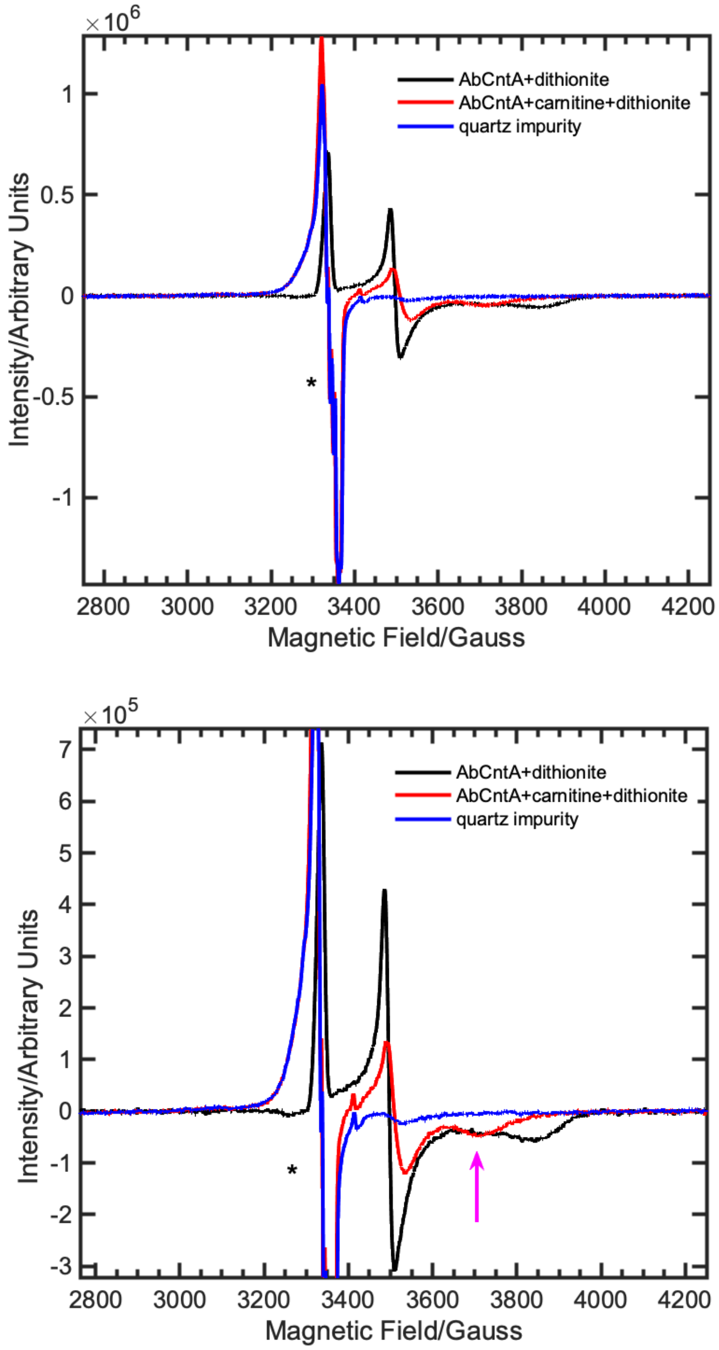

Figure S10. narrow-sweep, cw-EPR spectra of dithionite reduced, "as-isolated" *Ab*CntA in the presence (red trace) and absence (black trace) of the substrate, carnitine show the formation of one-electron reduced, Rieske, $[2Fe\text{-}2S]^{+1}$ EPR signals. The blue traces and asterisk marks in the top and bottom panels indicate the EPR signal arising from the quartz-impurity in the EPR tube. The magenta upward arrow indicates presence of a second EPR active species when *Ab*CntA is reduced with dithionite in the absence of carnitine (bottom; black trace), plausibly arise from the mono-nuclear, catalytic iron active-site. *Conditions* – as described in the experimental section.

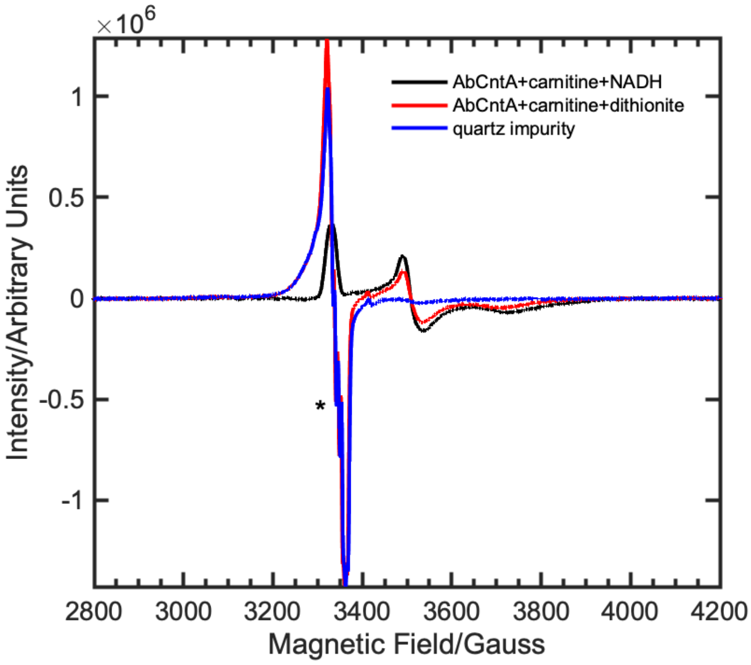

Figure S11. narrow-sweep, cw-EPR spectra of dithionite reduced, "*Ab*CntA+carnitine" (red trace) and photoactivated, "*Ab*CntA+carnitine+NADH" show the formation of similar/identical, one-electron reduced, Rieske, $[2Fe\text{-}2S]^{+1}$ EPR signals in both samples. The blue trace and asterisk mark indicate the EPR signal arising from the quartz-impurity in the EPR tube. *Conditions* – as described in the experimental section. If the intense, derivative like EPR signal observed at g = 2 (Figures, S10 and S11; blue trace and asterisk mark) is due to the presence of equilibrium between [$S_2O_4^{2-}$ & 2 SO_2^-] ions (monomeric, SO_2^- is paramagnetic; *J. Phys. Chem.,* **1959**, 63, 302; *Trans. Faraday Soc.,* **1969**, 65, 496-502), one would have expected to observe the same signal in the "*Ab*CntA-WT+dithionite" sample (Figures S9 and S10; black traces). The absence of this signal in "*Ab*CntA-WT+dithionite" rules out that the EPR signal at g = 2 signal was not due to dithionite.

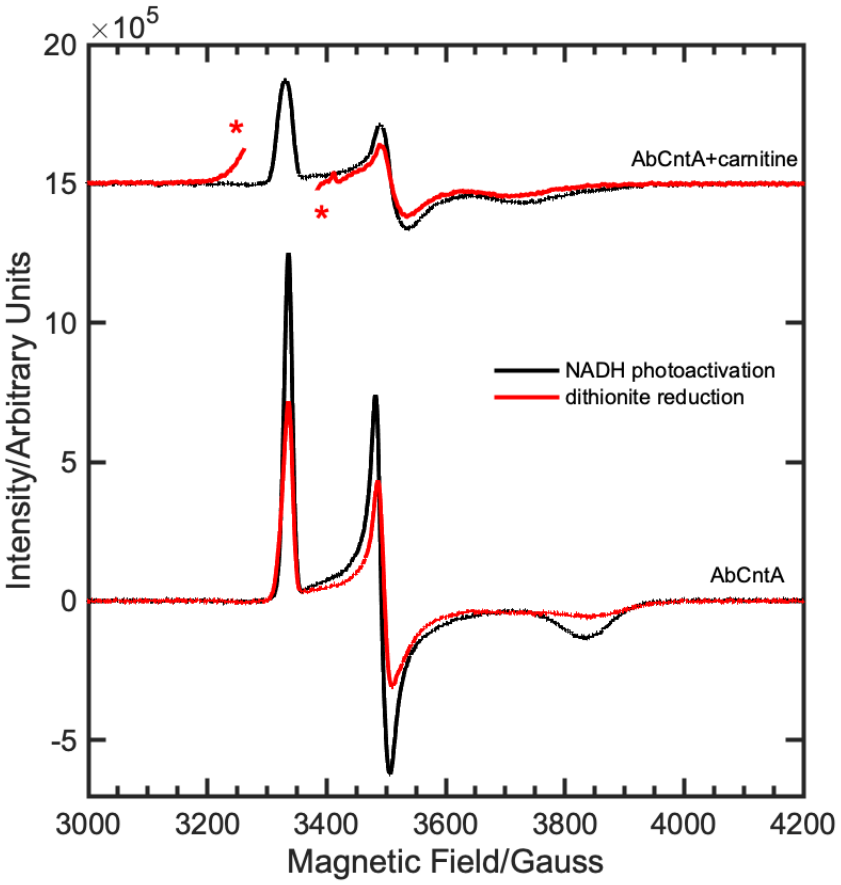

Figure S12. narrow-sweep, cw-EPR spectra of dithionite reduced (red traces) and NADH-photoactivated (black traces), "*Ab*CntA" in the presence (top) and absence (bottom) of carnitine show the formation of, one-electron reduced, Rieske, $[2Fe\text{-}2S]^{+1}$ EPR signals in both samples. The two red asterisk marks indicate that the strong EPR signals arising from the quartz-impurity in the EPR tube (see Figure S11) are truncated in this region to show the effective reduction by NADH-photoactivation process. *Conditions* – as described in the experimental section.

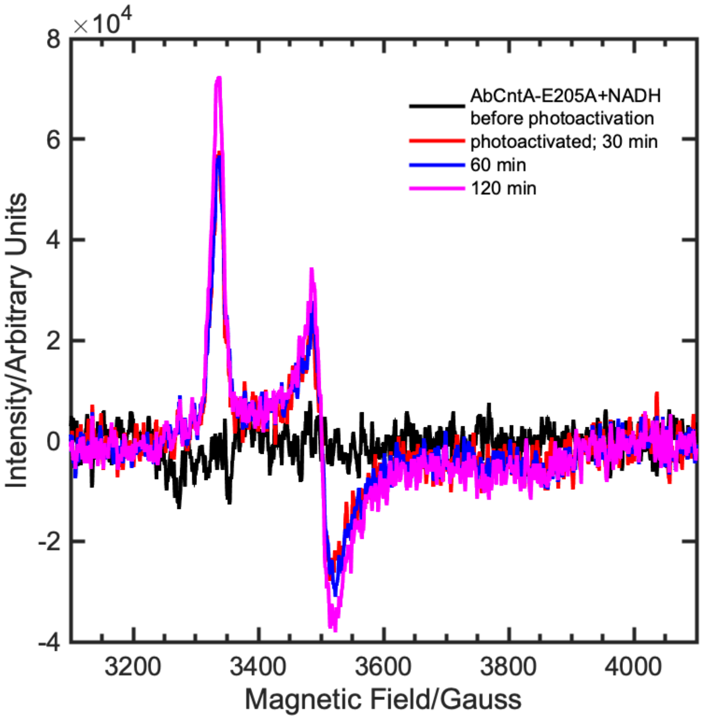

Figure S13. cw-EPR spectra of the '*Ab*CntA-E205A+NADH', following photoactivation using blue-light, 365 nm for various time scale. The spectra were measured as a frozen solution at 20 K. *Conditions* – as described in the experimental section.

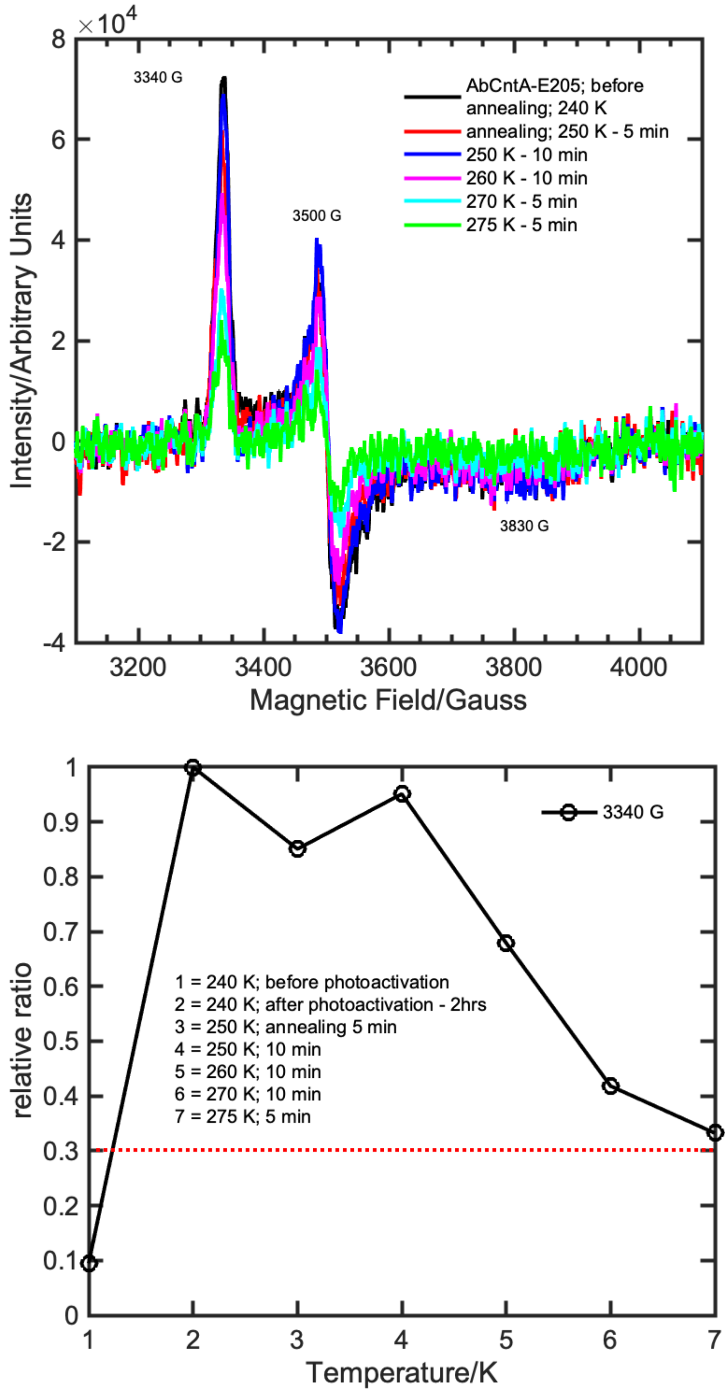

Figure S14. (top) EPR spectral changes observed when "*Ab*CntA-E205A+NADH" was annealed at the specified temperatures/times, following photoexcitation using 365 nm, blue-light for 120 minutes. (bottom) All the spectra were normalised relative to the EPR intensity observed at 240 K (3340 G) after photoactivation of NADH for 120 minutes. The comparison of relative EPR signal of "*Ab*CntA-E205A+NADH" observed at 275 K (~ 30 %) to that of "*Ab*CntA-WT +NADH" at 270 K (Figure S7; ~ 80 %), suggest that the bridging glutamate plays a critical role in stabilising the one-electron reduced, Rieske centre in the absence of carnitine. *Conditions* – as described in the experimental section.

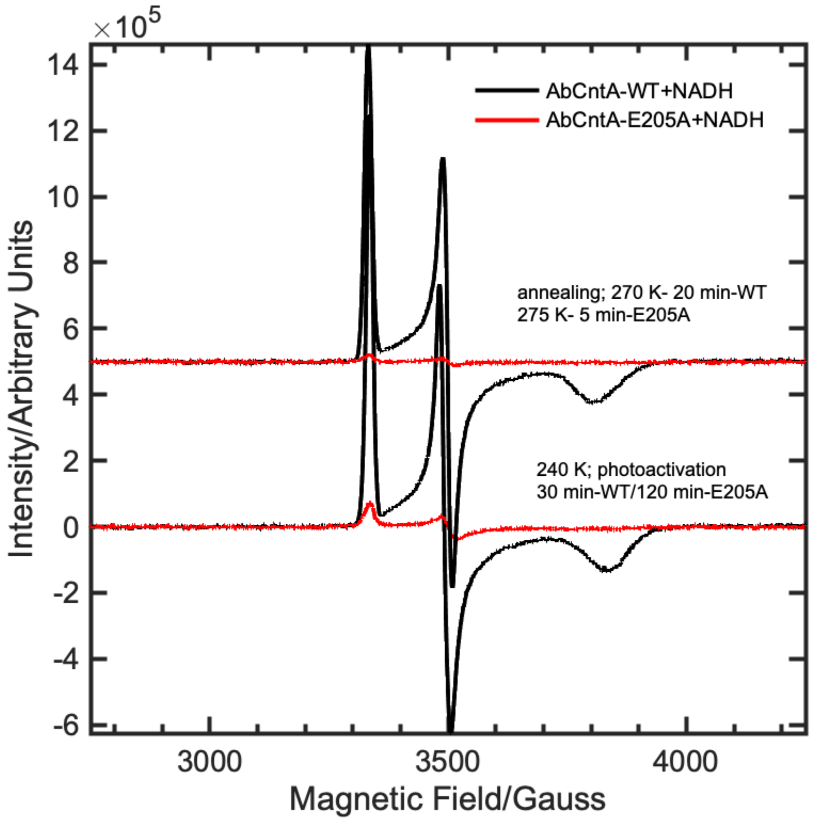

Figure S15. Comparisons of narrow-sweep, cw-EPR spectra of photoactivated, "*Ab*CntA-WT+NADH" (black traces; 30 min) and "*Ab*CntA-E205A+NADH" (red traces; 120 min) before (bottom) and after (top) annealing at 270 K (*Ab*CntA-WT+NADH – 20 min)/275 K (*Ab*CntA-E205A+NADH; 5 min). All EPR spectra were measured as a frozen solution at 20 K. The observation of significantly reduced catalytic activity for the "*Ab*CntA-E205A" mutant is likely due to the disruption in the electron transfer process and its incompetence to stabilise the one-electron reduced centre as shown in the comparison. *Conditions* – as described in the experimental section.

AUTHORS CONTRIBUTION

MS designed and conceived experiments, performed all EPR measurements and simulations, collated, analyzed and interpreted data, wrote the manuscript. MQ prepared all samples, and managed all experimental aspects of the project. YC, AC and TB secured funding and directed the overall project. All authors commented on the final manuscript.

REFERENCES

1. Y.Zhu, E. Jameson, M. Crosatti, H. Schafer, K. Rajakumar, T. D. H. Bugg and Y. Chen; *Proc. Natl. Acad. Sci.,* 2014, **111**, 4268–4273.
2. https://www.thorlabs.com/drawings/3498fd12177ef95e-F487ADAC-E14A-7416-E0B8341C5B6029A3/M365L2-SpecSheet.pdf
3. S. Stoll and A. Schweiger, *J. Magn. Reson.*, 2006, **178**, 42–55.
4. M. Massmig, E. Reijerse, J. Krausze, C. Laurich, W. Lubitz, D. Jahn and J. Moser; *J. Biol. Chem*, 2020, **295**, 13065-13078.
5. R. Davydov, T. M. Makris, V. Kofman, D. E. Werst, S. G. Sligar and B. M. Hoffman, *J. Am. Chem. Soc.*, 2001, **123**, 1403–1415.
6. T. M. Hedison, M. Shanmugam, D. J. Heyes, R. Edge and N. S. Scrutton, *Angew. Chem. Int. Ed.,* 2020, **59**, 13936-13940.
7. R. Davydov, A. A. Gilep, N. V. Strushkevich, S. A. Usanov and B. M. Hoffman, *J. Am. Chem. Soc.,* 2012, **134**, 17149-17156.
